# Social inequalities in mental health in Norway: possible explanatory factors

**DOI:** 10.1186/1475-9276-7-27

**Published:** 2008-12-24

**Authors:** Odd Steffen Dalgard

**Affiliations:** 1 National Institute of Public Health, Division of Mental Health, Sandakerveien 24 B, P.O. 4404 Nydalen, 0403 Oslo, Norway

## Abstract

**Background:**

It is well known that there is a social gradient in mental health, the prevalence of mental disorders stepwise increasing by lower social status. The reason for this, however, is not clear, and the purpose of the present study was to explore possible mediating factors between social status and mental health.

**Methods:**

The study has a cross-sectional design, and was based on a nationwide survey in Oslo, Norway, counting 12 310 people in the age of 30–60 years. Immigrants from non-western countries were excluded. Socio-demographic data were gathered from existing registers, whereas data on health, psychosocial variables and life style were gathered by structured interview. As indicator of mental health was used a 10-items version of Hopkins Symptom Checklist, measuring psychological distress. Measures of general self-efficacy and sense of powerlessness was used as indicators of control of own life situation.

**Results:**

A strong social gradient in mental health was found, the prevalence of psychological distress increasing by decreasing social status. Psychosocial factors, including self-efficacy, sense of powerlessness, control of work, social support and negative life events, in particular economic problems, as well as life style factors (physical exercise, BMI, smoking) and somatic health, likewise showed a social gradient, all risk factors increasing by decreasing social status. When adjusting for the risk factors in multivariate statistical analyses, the social gradient in mental health was eliminated. Low self-efficacy and sense of powerlessness emerged as important explanatory factors, alongside with poor social support, economic problems, smoking and somatic disorder.

**Conclusion:**

Both individual characteristics, supposedly linked to the personality, like low self-efficacy, and factors related to the actual life situation, like economic problems and a feeling of powerlessness, contribute to the social gradient in mental health, and both aspects should be addressed in preventive work.

## Background

Starting with the early findings of Faris and Dunham [1] there has been a great number of studies showing an inverse relationship between socio-economic status and mental disorders [2,3]. These findings refer to psychotic as well as non-psychotic disorders [4,5], and are repeated in different Western countries. With respect to the causes of the social gradient in mental disorders, there are two main hypotheses: the selection hypothesis and the social causation hypothesis. The first implies that the low socioeconomic status is a consequence of the disorder, or by personality characteristics predisposing for mental disorder, whereas the second hypothesis implies that low socioeconomic status causes a life situation which in itself increases the risk of developing mental disorder. This does not mean that the selection hypothesis is unrelated to social conditions, as early deprivation and social learning may contribute substantially to personality problems, which in turn leads to social failure. The point is that according to the selections hypothesis, the problems occur before the individual enters a certain social position. Whereas the selection hypothesis seems to be most relevant for schizophrenia, the social causation hypothesis seems to be more relevant for major depression (excluding bipolar disorder), non-psychotic disorders and psychological distress [4]. Among the non-psychotic disorders, however, there seems to be different mechanisms for different diagnoses, social causation being most important for anxiety and depression, and selection being most important for antisocial disorders [6]. As psychological distress, measured by symptoms of depression and anxiety, is the measure of mental health in the present study, on should expect social causation to be most important in this case.

Given that social causation explains at least some of the social gradient in mental disorder, the question is how. What are the mechanisms linking low socio-economic status to mental disorders? Several suggestions have been put forward. According to the "neo-material" hypothesis, the deprivation of material goods, like owing a care or a house, in itself represent a burden on mental health. Some evidence seems to support this hypothesis. There seems, however to be stronger evidence for the "psychosocial hypothesis", suggesting that factors like perceived job demands, perceived financial hardship, lack of social support and negative life events are mediators between socioeconomic status and mental health [7,8].

Lack of control over own life has been suggested as a crucial factor in explaining the high level of mental disorders in people with low socio-economic status, "control" being measured as "sense of mastery" [9,10], "self-efficacy" [11], lack of control of work [8], or other related measures. The perception of powerlessness and lack of control is then conceived as a stressor, which may influence somatic as well as mental health [12,13]. To which extent low sense of control, i.e. low self-efficacy, represents a personality trait caused by learning in early life, or is a psychological state caused by a situation of social deprivation and frustration, is a subject for discussion [14]. Obviously only the last interpretation is consistent with the social causation hypothesis, whereas the first implies that selection plays an important role for the social gradient in health. It is suggested that the explanation of social inequality in health by differences in self-efficacy, understood as a personality characteristic, represents a form of psychological reductionism, transforming social and political problems into individual psychological problems [15].

To the extent psychosocial factors may act as mediators between socio-economic status and mental health, it is a need to investigate which factors are most important, and how they interact. For this purpose, the present study will include a number of possible mediators, both psychosocial and life-style related, and also somatic health. Among the psycho-social factors, control of own life will be shown special interest, and be measured by two different tests and by a question on control of work.

### Hypotheses

There is a social gradient in psychological distress, and there is likewise a social gradient in psychosocial as well as life style related risk factors. When controlling for the risk factors, the social gradient in psychological distress will be reduced or eliminated. In the multivariate analysis lack of control of own life emerges as the most important mediating factors.

## Methods

### Sample

The Oslo Health Survey was conducted during 2000–01 as collaboration between the Norwegian Institute of Public Health, the University of Oslo and Oslo Municipality [16]. The population was all inhabitants in Oslo aged 30, 40, 45, 60 and 75 years, in total 40 888 persons. The response rate was 46%, yielding a study sample of 18 770. The response rate was positively associated with education, income, age, female gender, married and born in a Western country. However, when studying the attrition bias by linking register-based data from Statistics Norway of demographics, lifestyle and social security grants to the whole study population, the social gradient in health by different socio-economic variables seemed unbiased [16]. For the purpose of the present study with focus on the working population, the age group 30 – 60 years was chosen. Because of the special social situation of immigrants from low-income countries, only persons born in Norway, Western Europe and America were included, which reduced substantially the proportion with low socioeconomic status compared to the total sample. This left us with a study sample of 12 310. In this sample the response rate for each age group was as follows: Men: 30 years (32.2%), 40 years (37.6%), 45 years (39.9%), 59.60 years (53.4%). Women: 30 years (39.9%), 40 years (50.1%), 45 years (53.5%), 59–60 years (57.3%).

### Variables

#### Psychological distress

A 10-items abbreviation of Hopkins Symptom Checklist [17] was used as measure of psychological distress. Each item was rated on a scale of 1 (not at all) to 4 (extremely) during the past week. In contrast to the 25 items Hopkins Symptom Checklist (HSCL-25), where symptoms can be subdivided into depression and anxiety categories [18], the HSCL-10 provides a measure of general psychological distress. The internal consistency of the scale was high in the sample (Chronbach's alpha = 0.89). The scale is used as a continuous variable and a categorical variable with cut off point 1.85 [17].

#### Social status

The classification of social status was based on register information on education, income and self-reported occupation. Level of education was split in five categories: primary [1] secondary [2] post-secondary [3] first stage tertiary [4] and second stage tertiary [5]. Income was also split in five categories: < 100 000 [1] , 100–200 000 [2], 200 – 300 000 [3], 300–400 000 [4] and 400 000 + [5], all in NOK. Occupations were grouped according to the fivefold version of Eriksson & Goldthorp's scheme [19] giving five hierarchical levels, unskilled manual [1] , skilled manual [2] , routine non-manual and managers in small firms [3], lower grade professionals, owners of large firms [4], high grade professionals, administrators and managers [5]. A summary index, ranging from 3 to 15, was constructed by combining data on education, income and occupation, and divided into five groups of about equal size. This index was reduced into three groups when dealing with separate somatic disorders.

#### Social support

*Social support *was measured by two questions, one about *number of close confidants*, and the other about perceived interest from others: *How great an interest do people take in what you are doing? (A lot of interest/some interest//uncertain/little interest/no interest)*.

#### Negative life events

To measure negative life events, an inventory of threatening experiences was used in the screening questionnaire [20]. This inventory consists of 12 questions, but in the present study the question on accident/serious somatic illness was omitted to avoid inbuilt association between life events and somatic disorder.

#### Perceived control

Perceived control was measured by two instruments and one single question on perceived work conditions. The instruments were *generalized self-efficacy *[21], with 10 items, and the *powerlessness *scale with 8 items from the more extensive Empowerment scale developed by Rogers et al. [22]. The two scales differ considerably in their content. Whereas the generalized self efficacy scale measures the confidence in being able to control challenging environmental demands by means of taking adaptive actions, the powerlessness scale measures the sense of power in a community context.

Examples of questions from the *self-efficacy *scale are:

I always manage to solve difficult problems if I try hard enough.

I am confident that I could deal efficiently with unexpected events.

Thanks to my resourcefulness, I know how to handle unforeseen situations.

Examples of questions from the *powerlessness *scale are:

You cannot fight city hall.

When I am unsure about something, I usually go along with the group.

Experts are in the best position to decide what people should do and learn.

In Principal Component Analysis with two factors extraction (Oblimin with Kaiser Normalization), self efficacy and powerlessness emerged as separate factors, but with two items in the powerlessness scale (feeling without power and feeling alone) loading about equally high on the two factors. Cronbach's alpha of self-efficacy was 0.89 and of powerlessness 0.64.

When adding the HSCL-10 items to the Principal Component Analysis in a three factor extraction, psychological distress, self-efficacy and powerlessness emerged as separate factors. Two items, however, the same as mentioned above, loaded higher on the psychological distress factor than the powerlessness factor. To avoid associations due to overlapping definitions of concepts, the main analyses are also repeated with a powerlessness scale where these two items are excluded.

The question on perceived *control at work *was: *Can you yourself decide how your work should be organized (not at all/to a small degree/largely/I decide myself)*.

#### Life style

Four factors of life style were chosen: Physical exercise, body mass index (BMI), smoking and consumption of alcohol.

*Physical exercise *was measured by the following question:

*Describe the extent of movement and bodily exertion in your spare time. (Read, watch TV or other sedentary activity*[1]*/walk, cycle or move about in some other way at least 4 times per week *[2]*/take part in physical exercise/sport, do heavy gardening work, at least 4 times a week *[3]*/exercise hard or take part in competitive sport regularly and several times a week *[4]*)*.

Smoking was measured by the question:

*Have you smoked/do you smoke daily? (never *[1]*, yes earlier*[2]*, yes now *[3]*)*

#### Somatic health

Information on somatic health was based on the question:

Do you have any of these illnesses, or have you suffered from them in the past? Yes/no.

The following somatic illnesses were included as separate response categories:

Asthma, chronic bronchitis/emphysema, diabetes, osteoporosis, fibromyalgia/chronic pain syndrome, cardiac infarction, angina pectoris, stroke/cerebral haemorrhage.

Skin disease was measured by a detailed questionnaire about various skin symptoms, which have been transformed into a validated scale [23]. In the present study the cut off point for caseness was 1.5 on the scale, indicating clinical skin illness.

Cardiac infarction, angina pectoris, and stroke/cerebral haemorrhage were combined into one dichotomous variable, cardiovascular disorder, with the categories "none" or "any".

The information on somatic health was then based on seven mainly chronic illnesses, and summarized into an index of number of illnesses reported.

### Statistical analysis

Gender differences in the distribution of indicators of social status were tested by Pearson's chi-square tests (table 1). Logistic regression analysis was used to test the association between social status and psychological distress, with HSCL cut of point 1.85, with adjustment for age (table 2). Differences in mean scores on risk factors between status groups were tested by analysis of variance (table 3). Logistic regression analysis was finally used to estimate the association between social status and psychological distress, when adjusting for different combinations of risk factors (table 4). Data were analyzed by Statistical Package for Social Sciences (SPSS), version 12 for Windows, version 12.

**Table 1:** Sample distribution of indicators of social status by gender.

		Men	Women	Sign.
Education	Primary	557 (12.2)	800 (13.7)	
	Secondary	912 (19.9)	1055 (18.1)	
	Post-secondary	487 (10.6)	758 (13.0)	***
	First stage tertiary	1021 (22.3)	1306 (22.4)	
	Second stage tertiary	1604 (35.0)	1908 (32.7)	
	
	Total	4581 (100.0)	5827 (100.0)	

Income NOK	< 100 000	171 (3.8)	509 (9.0)	
	100–200 000	422 (9.3)	1282 (22.6)	
	200–300 000	1215 (26.8)	2494 (43.9)	***
	300–400 000	1348 (29.7)	992 (17.5)	
	400 000 +	1381 (30.4)	403 (22.6)	
	
	Total	4537 (100.0)	5680 (100.0)	

Occupation	Unskilled manual	364 (7.4)	203 (3.4)	
	Skilled manual	384 (7.8)	109 (1.8)	
	Non-manual	1969 (40.0)	4039 (68.5)	
	Lower service	617 (12.5)	539 (9.1)	***
	Higher service	1598 (32.3)	1003 (17.0)	
	
	Total	4923 (100.0)	5893 (100.0)	

Social status combined index	I (low)	727 (17.9)	1112 (22.3)	
	II	582 (14.3)	1222 (24.5)	
	III	629 (15.5)	1260 (25.3)	***
	IV	892 (22.1)	737 (14.8)	
	V (high)	1225 (30.2)	654 (13.1)	
	
	Total	4060 (100.0)	4985 (100.0)	

P < 0.05*

P < 0.01**

P < 0.001 ***

Absolute figures (percentages)

## Results

The distribution of social status by gender is shown in table 1.

For all indicators there was a significant gender difference. The women had lower education and income and smaller fraction of high-status occupations than the men.

### Social status and psychological distress

The association between social status and psychological distress is shown in table 2, where psychological distress is dichotomized with cut off point 1.85.

In both genders there was a significant social gradient, psychological distress increasing by lower social status.

**Table 2:** Association between social status and psychological distress (HSCL-10).

Social status	Men		Women	
	
	OR	95% CI	OR	95%CI
I	3.62	2.26–5.34	2.77	1.94–3.97
II	1.92	1.22–3.03	1.73	1.20–2.49
III	1.78	1.12–2.81	1.54	1.07–2.22
IV	1.07	0.67–1.70	1.14	0.75–1.74
V	Ref.			

Logistic regression, adjusted for age.

### Social status and risk factors

The distribution of risk factors (psychosocial factors and life style factors) by social status and gender is shown in table 3.

For most of the risk factors the values tend to increase, respectively decrease, by level of socio-economic status. For some factors, however, the pattern is less regular, but even then the values increase, respectively decrease, when comparing the average of the higher levels with the average of the lower levels. With respect to negative life events, seven out of eleven items showed a social gradient, two items a tendency in the same direction, and only two items no association with social status. Economic problems showed the strongest social gradient, 9.9% reporting problems in the lowest status group, against 1.8% in the highest group.

To control for inverse causality, i.e. that the social gradient in risk factors was caused by the social gradient in psychological distress, and not the other way around, logistic regression analysis was carried out when adjusting for psychological distress. This did not substantially alter the pattern. Irrespective of psychological distress, the risk factors showed a social gradient.

**Table 3:** Risk factors by social status and gender.

	Social status	Contr. work	Power- less- ness	Self-efficacy	Confidants	Soc. supp.	Life events	Exercise	BMI	Smoking	Som. disorder
M	I	2.73	2.27	3.04	8.03	3.58	0.90	2.01	26.92	2.16	0.36
	II	2.98	2.14	3.10	8.57	3.84	0.71	2.11	26.82	1.96	0.29
	III	2.99	2.08	3.12	8.38	4.00	0.71	2.11	26.46	1.78	0.26
	IV	3.13	2.06	3.13	8.80	3.99	0.56	2.13	26.51	1.76	0.25
	V	3.18	2.00	3.17	8.68	4.12	0.60	2.13	26.25	1.61	0.23
	
	Sign.	***	***	***		***	***	*	**	***	***

W	I	2.54	2.28	2.97	7.84	3.74	0.78	1.91	25.87	2.11	0.50
	II	2.74	2.14	3.00	8.60	3.96	0.79	1.96	25.26	1.89	0.39
	III	2.84	2.06	3.05	9.30	4.11	0.66	1.99	24.79	1.74	0.29
	IV	2.98	2.04	3.08	9.66	4.18	0.65	2.02	24.28	1.67	0.27
	V	3.09	2.00	3.16	10.36	4.21	0.61	2.03	24.36	1.55	0.32
	
	Sign.	***	***	***	***	***	**	***	***	***	***

Means, adjusted for age.

### Social status, risk factors and psychological distress

The associations between social status and psychological distress, when adjusting for age, gender and various combinations of risk factors is shown in table 4. (The figures for men and women were combined because of small gender differences).

**Table 4:** Associations between social status, risk factors and psychological distress (HSCL-10 > 1.85).

Social status and risk factors	Model 1	Model 2	Model 3	Model 4	Model 5	Model 6
Social Status						
I	3.10 (2.38–4.03)	1.33 (0.98–1.80)	2.51 (1.91–3.30)	2.52 (1.92–3.30)	2.08 (1.55–2.80)	0.92 (0.65–1.29)
II	1.84 (1.40–2.43)	1.13 (0.83–1.53)	1.61 (1.21–2.15)	1.57 (1.19–2.08)	1.50 (1.11–2.03)	0.89 (0.63–1.24)
III	1.65 (1.25–2.19)	1.31 (0.97–1.77)	1.59 (1.20–2.12)	1.57 (1.18–2.08)	1.54 (1.14–2.08)	1.23 (0.89–1.71)
IV	1.13 (0.83–1.54)	0.93 (0.67–1.29)	1.06 (0.77–1.46)	1.07 (0.78–1.46)	1.08 (0.77–1.51)	0.83 (0.58–1.19)
V	Ref.	Ref.	Ref.	Ref.	Ref.	Ref

Control work		0.90 (0.79–1.02)				1.00 (0.87–1.15)
Powerlessness		3.89 (3.13–4.82)				2.76 (2.16–3.52)
Self-efficacy		0.28 (0.23–0.35)				0.28 (0.22–0.37)

Confidants			0.93 (0.92–0.95)			0.94 (0.92–0.96)
Social support			0.74 (0.69–0.80)			0.90 (0.81–0.99)

Economic problems.				4.64 (3.69–5.85)		2.91 (2.12–3.98)

Exercise					0.76 (0.67–0.87)	0.95 (0.82–1.10)
BMI					1.02 (1.00–1.04)	1.00 (0.98–1.03)
Smoking					1.37 (1.23–1.51)	1.43 (1.27–1.61)
Som. disorder					1.95 (1.76–2.16)	1.72 (1.52–1.96)

Model 1: Adjusted for age and gender

Model 2: Adjusted for age, gender, control of work, powerlessness and self-efficacy

Model 3: Adjusted for age, gender, confidants and social support

Model 4: Adjusted for age, gender and economic problems

Model 4: Adjusted for age, gender, exercise, BMI, smoking and somatic disorders

Model 5: Adjusted for age, gender and all risk factors

Logistic regression. Adjustment for age, gender and various combinations of risk factors.

Model 1 shows the social gradient in psychological distress, when adjusting only for gender and age. When adjusting for control of work, powerlessness and self-efficacy (model 2), the social gradient in psychological distress was reduced below the level of statistical significance. When alternatively adjusting for number of confidants and social support (model 3), there was also a reduction in the social gradient of psychological distress, but weaker than in model 2. Also adjustment for economic problems (model 4), reduced the social gradient in psychological distress, but again to a lesser extend than when adjusting for control at work, powerlessness and self-efficacy. When adjusting for life style factors (model 5), the social gradient in psychological distress was again reduced, but not as strongly as in model 2. In the full model (model 6) the social gradient in psychological stress was eliminated, powerlessness, self-efficacy, economic problems, smoking and somatic disorder standing out as independent predictors of psychological distress.

When doing this analysis with the shortened powerlessness scale (because of two overlapping items between psychological distress and the full powerlessness scale), the results were somewhat changed. In model 2, the odd's ratio of powerlessness was reduced to from 3.98 (3.13–4.82) to 1.32 (1.11 – 1.58), with small changes in the odd's ratios of control of work and self-efficacy. The odd's ratios of social status were less reduced than when adjusting for the full powerlessness scale, the figures for the respective status groups now being: I: 1.67 (1.23 – 2.25). II: 1.28 (0.95 – 1.72). III: 1.38 (1.02 – 1.85). IV: 0.96 (0.69 – 1–33). In spite of these changes, in the full model (model 6) the social gradient of social status was eliminated.

## Discussion

### Main findings

There was a social gradient in psychological distress in both genders, and a stepwise reduction in the odd's ratios of social status by increasing status. With some exceptions, there was also a social gradient in life style factors and psychosocial factors. For men, there was a clear gradient in somatic disorders, whereas the pattern was less clear in women. In women there was a tendency that the highest status group (V) had more somatic disorders than group III and IV. When adjusting for all risk factors, the social gradient of psychological distress was eliminated, powerlessness, self-efficacy, economic problems, smoking and somatic disorder emerging as independent risk factors of psychological distress. When using a shortened powerlessness scale, with elimination of two items overlapping with the psychological distress factor, the importance of powerlessness was reduced, whereas the other associations were essentially unchanged.

### Methodological issues

The main weakness of the study is the cross-sectional design, which makes it impossible to draw certain conclusions on causal relationships. It is difficult to decide what came first, the social status, the suggested risk factors or the psychological distress. It could for instance be that the social gradient in risk factors did not cause the social gradient in somatic disorders, but conversely, that the gradient in risk factors was a consequence of the social gradient in psychological distress. To some extent, however, this possibility has been controlled for, since the social gradient in risk factors persisted even when adjusting for psychological distress. Still the possibility of reversed causality exists, i.e. psychological distress causing lowered social status rather than the other way around. Only longitudinal studies can give answers to these questions.

For the psychosocial risk factors, in particular powerlessness and self-efficacy, the possibility also exists that the association with psychological distress is due to overlapping in the definition of concepts, and not the one causing the other. This is, however, to some extent controlled for in factors analysis, indicating that self-efficacy and psychological distress are to distinct concepts. With respect to powerlessness, however, there is some overlapping with psychological distress, two items of the powerlessness scale loading higher on the psychological distress factor than the powerlessness factor. When these two items were excluded, the association between the two factors was reduced, and powerlessness as mediating factor between social status and psychological distress likewise reduced. Hence among the psychosocial factors, self-efficacy and economic problems seem to be the most important mediators, whereas the seemingly strong effect of powerlessness is partly due to overlapping concepts.

The low response rate may question the representativeness of the sample. However, as earlier mentioned, the underrepresentation of the lower socio-economic groups did not seem to affect the social gradient in health. Even if the proportion with low education and low income is smaller in the present sample than in the total sample, because of the exclusion of immigrants from low-income countries, this should not affect substantially the relative figures as expressed in logistic regression analysis.

It is a strength of the study that it is based on a fairly big sample, making it possible to deal with a number of factors related to mental health, psychosocial as well as life style factors and somatic health. This allows for a holistic approach to health, not placing in mental health and psychosocial factors on the one side, and life style and somatic health on the other.

### Lack of control and mental health

It is in accordance with the hypotheses of the study that lack of control over own life situation appears as a major mediating factor between social status as psychological distress. Lack of control may influence psychological health by frustrating the need for autonomy and by inducing a state of perceived powerlessness, which over time may lead to anxiety and depression.

To which extent this lack of control is a result of personality traits and lack of individual coping resources, or is rather a reaction to a difficult life situation, cannot be answered by the present study. However, data indicate that both individual factors and situational factors are of importance. On the one side, the strong association between psychological distress and self-efficacy, which at least to some extent is supposed to be linked to personality traits [11], indicates that selection mechanisms contributes to the social gradient in mental health. On the other side, the association between psychological distress and powerlessness, which is probably more linked to the actual life situation than lack of self-efficacy, and economic problems, points towards situational factors.

Given that both individual factors and situational factors contribute to the social gradient in mental health, both aspects has to be addressed in preventive work. Without doing something about the unequal distribution of economic resources, even in a welfare state like Norway, one is not likely to succeed. On the other side, without programs aiming at the strengthening of individual coping abilities, in the terms of "empowerment", improving the economic situation may not lead to lasting results.

## Competing interests

The author declares that they have no competing interests.
